# Exploring the absence of LMP1-XhoI deletion in nasopharyngeal carcinoma patients: A genetic perspective

**DOI:** 10.1016/j.jtumed.2025.11.009

**Published:** 2025-12-15

**Authors:** Yaqeen Rjoub, Mai Abusalah, Aseel Al-Hussein, Khaled Al-Qaoud, Anwar Rjoop, Yasmin AlSaidat, Moayad A. Rjoub, Manal Abusalah, Naveed Ahmed

**Affiliations:** a Department of Medical Lab Sciences, Zarqa University, Zarqa, Jordan; b Department of Medical Laboratory Sciences, Faculty of Allied Medical Sciences, Al-Ahliyya Amman University, Jordan; c Department of Biological Sciences, Al-Yarmouk University, Irbid, Jordan; d Department of Pathology and Microbiology, Faculty of Medicine, Jordan University of Science and Technology, Irbid, Jordan; e King Abdullah University Hospital, Irbid, Jordan; f Department of Histopathologic and Hematopatgologist, Princess Iman Laboratory for Science and Research, Royal Medical Services, Amman, Jordan; g King Hussein Medical Center, Amman, Jordan; h Department of Special Surgery and Urology, Faculty of Medicine, Jordan University of Science and Technology, Irbid, Jordan; i Department of Medical Microbiology and Parasitology, School of Medical Sciences, Universiti Sains Malaysia, Kelantan, Malaysia; j Department of Assistance Medical Sciences, Applied College, University of Tabuk, Tabuk, KSA

**Keywords:** Cancer, EBV, Molecular study, Mutations, Oncovirus, دراسةجزيئية, سرطان, طفرات, فيروس, فيروسمُسرطِن

## Abstract

**Background:**

Nasopharyngeal carcinoma (NPC) is the most common type of head and neck cancer, and its development is closely linked to Epstein–Barr virus (EBV). Notably, changes in the viral Latent Membrane Protein 1 (LMP-1) gene, such as the absence of the Xhol restriction site, contribute to its oncogenic properties. This study was to explore the connection between the XhoI site and NPC progression, with the goal of enhancing early diagnosis and treatment strategies.

**Methods:**

Formalin-fixed paraffin-embedded (FFPE) tissues from NPC patients were collected, and using restriction fragment length polymorphism (RFLP-PCR), variants of the EBV LMP-1 XhoI gene were identified, followed by amplification of the BamHI-W gene.

**Results:**

EBV DNA was detected in all NPC tissue samples via amplification of the BamHI-W region, confirming widespread EBV association in the Jordanian cohort. Wild-type (WT) XhoI variant was identified in 82.86 % of NPC cases, whereas no samples exhibited the mutant-type (MT) XhoI variant, as substantiated by Sanger Sequencing of representative cases. Statistical analysis revealed no significant correlation between the presence of WT XhoI and variables such as patient sex, age, tissue origin, NPC subtype, or disease stage. Notably, most samples from stage III/IV harbored the WT XhoI variant. Overall, BamHI-W gene detection proved to be a more consistent molecular marker than LMP-1 XhoI in this population, and no relationship was established between LMP-1 XhoI mutation and NPC development

**Conclusion:**

These findings primarily highlight the epidemiological association between EBV and NPC in Jordanian patients, rather than demonstrating immediate clinical applicability for screening or early diagnosis. The absence of the LMP-1 XhoI deletion in this cohort underscores significant geographic and ethnic heterogeneity in the prevalence of this genetic alteration among NPC patients.

## الملخص

**أهداف البحث:**

يُعَدّ سرطان البلعوم الأنفي أكثر أنواع أورام الرأس والعنق شيوعًا، ويُعزى تطوره بشكل وثيق إلى فيروس إبشتاين–بار. وتُعَدّ التبدلات في مورثة البروتين الغشائي الكامن-1 للفيروس، مثل غياب موقع التقطيع بالإنزيم النوكي المتخصّص (XhoI)، من العوامل التي تسهم في الخاصية المسرطِنة لهذا البروتين. تهدف هذه الدراسة إلى استكشاف العلاقة بين موقع XhoI وتطور سرطان البلعوم الأنفي، بهدف تعزيز إمكانات التشخيص المبكر وتحسين الاستراتيجيات العلاجية.

**طرق البحث:**

جرى إعداد أنسجة مُثبّتة بالفورمالين ومُضمّنة بالبارافين من مرضى سرطان البلعوم الأنفي، وتم تحديد متحورات مورثة البروتين الغشائي الكامن-1 اعتمادًا على تقنية تضخيم تمييز طول الشدف المُقيَّدة، تلتها عملية تضخيم مورثة BamHI-W.

**النتائج:**

تم كشف المادة الوراثية لفيروس إبشتاين–بار في جميع عينات سرطان البلعوم الأنفي عبر تضخيم منطقة BamHI-W، مما يؤكد الارتباط الواسع للفيروس في العيّنة الأردنية. وظهر المتحوّر السنخي (النوعي) لموقع XhoI في 82.86٫٪ من الحالات، في حين لم تُرصد أي حالة للمتحور الطافر وفقًا لنتائج التسلسل الجيني بطريقة سانغر للعينات الممثِّلة. ولم تُظهر التحليلات الإحصائية أي علاقة ذات دلالة بين وجود المتحوّر السنخي ومُتغيّرات مثل جنس المريض، والعمر، ومصدر العينة النسيجية، والنمط النسيجي لسرطان البلعوم الأنفي، أو مرحلة المرض. ولوحظ أن معظم العينات في المرحلتين الثالثة والرابعة تحمل المتحور السنخي. وبشكل عام، تبيّن أن كشف مورثة BamHI-W يُعدّ علامة جزيئية أكثر ثباتًا من موقع XhoI للبروتين الغشائي الكامن-1 في هذه الفئة السكانية، ولم تُثبَت أي علاقة بين طفرة موقع XhoI وتطور سرطان البلعوم الأنفي.

**الاستنتاجات:**

وتُشير هذه النتائج بشكل رئيس إلى الارتباط الوبائي بين فيروس إبشتاين–بار وسرطان البلعوم الأنفي لدى المرضى الأردنيين، بدلًا من تقديم قيمة سريرية مباشرة في الفحص التحري أو التشخيص المبكر. كما يُبرز غياب حذف موقع XhoI في هذه الفئة السكانية وجود اختلافات جغرافية وعرقية واضحة في معدل انتشار هذا التبدل الجيني بين مرضى سرطان البلعوم الأنفي. ويتوافق ذلك مع ما ورد في التحاليل التلوية والدراسات السكانية واسعة النطاق، التي تُظهِر تباينًا ملحوظًا في تعدد أشكال موقع XhoI لمورثة البروتين الغشائي الكامن-1 عبر مناطق وعرقيات مختلفة عالميًا.

## Introduction

One of the main causes of significant death worldwide is cancer. About 1.5 % of all cancer cases globally are Epstein–Barr virus (EBV)-associated malignancies, which also account for 1.8 % of cancer-related fatalities. Numerous human cancers, including lymphoid and epithelial cancers, such as nasopharyngeal carcinoma (NPC), are associated with EBV.^1^

NPC is a malignant cancer that is more common in southern China but less common in Jordan.^2^ It exhibits a unique geographic and ethnic distribution, with incidence rates varying up to 25-fold between high and low NPC regions.^3^ Southern China has the highest incidence rate, particularly among Cantonese populations.^4^ About 30 cases per 100,000 people are observed yearly. Southeast Asian nations and the Eskimos are two more regions with a high incidence rate.^5^ NPC is the fourth most common tumor in Hong Kong.^6^ NPC is uncommon in the USA and Western Europe; fewer than 0.5–1 occurrences per 100,000 people are reported yearly.^6^ Over decades, this incidence has remained stable.^7^ North African^8^ and Arab reports indicate an intermediate to high risk.^9^ According to WHO data, it was reported that 45 people in Jordan in 2018 had NPC of both sexes, representing 0.6 % of all cancer types by the primary site.

NPC can be defined as a kind of head and neck cancer kind.^10^ NPC originates in the nasopharynx's epithelial cells. The epithelial cells of the nasopharynx are the source of NPC. The tumor point is usually seen at the Rosen Müller fossa, which is located inside the nasopharynx. The tumor then spreads into adjacent anatomical areas or organs.^11^ Though the incidence of NPC is thought to be low globally, it is associated with a poor prognosis that translates into a lower survival rate.^12^ It is well known that there is a correlation between EBV and NPC in a variety of risk populations.^13^

EBV is a common virus that causes latent infection and affects 95 % of people globally. Most of these diseases have no symptoms at all. However, non-small cell lung cancer (NPC), particularly the poorly differentiated NPC that is more prevalent in Southern China, has been connected to EBV.^14^ It is believed that somatic mutations in precancerous lesions and EBV infections interact strongly to affect the development of NPC.^15^ The expression of many latent EBV genes is one characteristic of latent EBV infection. EBV produces a latent gene product called latent membrane protein 1 (LMP 1). Apart from its usual carcinogenic properties in the transformation of murine fibroblasts, it is thought to be a significant oncoprotein since it can produce different phenotypic alterations in B cells and epithelial cells.^16^ The significance of LMP-1 in NPC carcinogenesis is demonstrated by the fact that 78 % of NPC samples express this protein.^16^ LMP-1 expression is linked to an increased risk of metastasis in NPC^17^ LMP-1 can be used as a rapid indicator of concealed metastases in main NPC. Even though EBV is linked to 90 % of NPC cases, the reported percentage of LMP-1 expression using current methods varies between 50 % and 80 %.^17^ The Xhol restriction site in the cytoplasmic N-terminal tail of the LMP-1 gene has been deleted due to a point mutation found at nucleotide position G169425T.^12^ A meta-analysis of 31 observational studies found that the XhoI polymorphism is present in about 77 % of samples from patients with NPC but not in samples from healthy people.^12^

Finding EBV genes involved in oncogenicity can be a very helpful diagnostic and prognostic tool, especially as latent EBV infection is strongly linked to the development of NPC. The current study examines whether tissue samples from NPC Jordanian patients include various genetic indicators, such as LMP-1 XhoI and BamHI-W. The current investigation aims to determine if the development of NPC in the Jordanian population is significantly correlated with the existence or expression of these biomarkers. A robust correlation may result in the creation of NPC diagnostic instruments and the identification of factors that predict the course of the illness, facilitating more focused and efficient therapeutic approaches.

## Materials and Methods

### Study population and sample collection

Seventy NPC formalin-fixed paraffin-embedded (FFPE) samples were obtained for this study from King Abdulla University Hospital (KAUH) and King Hussein Medical Center (KHMC). DNA was extracted from tissue samples embedded in paraffin. The clinical data were collected for each patient, including the type of NPC, age, the computed tomography (CT) scan for the stage of the disease, gender, and the last follow-up for the patient.

### DNA extraction

The FFPE sample block was cut using a microtome (Manual Rotary Microtome Cut 4060, SLEE Medical GmbH, Germany) into up to 8 sections that were 5–10 μm thick. A number of commercial extraction kits were used; however, the GeneAll® ExgeneTM FFPE Tissue DNA extraction kit (Gene All Biotechnology, Korea) produced the purest results and the highest DNA concentration. The manufacturer's instructions were followed when performing the DNA extraction process. The column was incubated for 1 min at room temperature before the final centrifugation at 14,000×*g* for 1 min. Using a spectrophotometer (PG/T60 UV–Visible Spectrophotometer, China), the amount of total DNA was measured and kept at −20 °C until it was needed.

### Polymerase chain reaction (PCR)

PCR amplification of the BamHI-W and LMP-1 XhoI gene regions was conducted using the Veriti heat cycler (Applied Biosystems, USA). As detailed in Table 1, target-specific sense and antisense primers (final concentration: 10 μM) were utilized, and reaction conditions were optimized for each amplicon. For BamHI-W, each 25 μl reaction contained 4 μl of extracted DNA, 2 μl of each primer, 12.5 μl of master mix, and 4.5 μl of nuclease-free water (NFW). Cycling condition consisted of an initial denaturation at 95 °C for 5 min, followed by 35 cycles of 30 s at 95 °C, annealing at 54 °C for 30 s, and extension at 72 °C for 5 min.

**Table 1:** The Primer Sequences and PCR Conditions Used in This Study.

Gene	Primer Sequences	Product size (bp)	Annealing Temp and Time	References
Xhol LMP-1	Forward X1.1 (5′-ATGGAACACGACCTTGAGAGG-3′) Reverse X1.2 (5′-AACAGTAGCGCCAAGAGCAG-3′)	Wild type (67 + 46) bp Mutant type 113 bp	58 °C for 30sec	17
BamHI-W	Forward (5′ AGTGGGCTTGTTTGTGACTTCA 3′) Reverse (5′GG ACTCCTGGCGCTCTGAT 3′).	191 bp	54 °C for 30sec	30
Bata actin	Forward (5′-TTCCTTCCTGGGCATGGAGT-3′) Reverse (5′-GCAATGATCTTGATCTTCATTG-3′)	201 bp	58 °C for 30sec	This study

For LMP-1 XhoI amplification, 6 μl of extracted DNA, 2 μl of each primer, 12.5 μl of master mix, and 3.5 μl of NFW were employed in a 25 μl reaction volume; cycling comprised an initial 5-min denaturation at 95 °C, followed by 35 cycles with an annealing temperature of 58 °C for 30 s. The housekeeping gene (β-actin) was amplified under conditions analogous to those used for LMP-1 XhoI, as indicated in Table 1. All primer sequences, product sizes, and annealing conditions are provided in Table 1.

### Enzyme digestion of XhoI PCR products

In this work, a PCR product was digested using the XhoI restriction enzyme (Clontech Takara Cellartis, Japan). The reaction mixture included 2 μl of the 1X (2M) buffer R, 2 μl of 2M XhoI enzyme, 10 μl of the purified PCR product, and 6 μl of nuclease-free water; the reaction mixture had a total volume of 20 μl. The reaction was heat-inactivated for 20 min at 50 °C following an overnight incubation period at 37 °C.

### Agarose gel electrophoresis

PCR products were examined before and after digestion using gel electrophoresis on a 3 % low-melting agarose gel (LE) (Cleaver Scientific, UK). The electrophoresis was conducted at 100 V for 50 min using the electrophoresis system (Thistle Scientific Ltd., Uddingston, Scotland). After electrophoresis, the gel was visualized using an ultraviolet transilluminator imaging system (Thistle Scientific Ltd., Uddingston, Scotland). To determine the molecular weight of the DNA fragments, a standard DNA ladder mix (50 bp and 100 bp) (GeneDireX, Taiwan) was used.

### Sequencing of DNA

To verify the existence of LMP-1 Xhol, six amplicons from NPC tissue DNA were chosen at random and sequenced using the BigDye® Terminator v3.1 sequencing kit and ABI PRISM® 377 Genetic Analyzer. To find any differences in the nucleotide sequence, the sequencing findings were then compared to the B95.8 prototype EBV genome (GenBank accessions no.: V01555.2, locations 169,474 bp to 168,163 bp).

### Statistical analysis

IBM SPSS (version 25) was used for statistical analysis after Microsoft Excel was used to compile the data. P-values less than 0.05 were regarded as statistically significant. The correlation between LMP-1 Xhol and clinical or demographic factors was investigated using the Fisher's exact test and Chi-square test.

## Results

### NPC general characteristics of the studied population

Seventy tissue samples in all were used in the investigation. According to the survey, the majority of NPC Jordanian patients (80.31 %) were men, with only 19.69 % being women. The patients were 51.1 ± 14.3 years old on average. According to the findings, the largest percentage (n = 39, 81.25 %) was recorded by the undifferentiated cell carcinoma (UC) type. Two instances (4.17 %) had different types (keratinizing squamous cell carcinoma (SCC) and basaloid squamous cell carcinoma), whereas seven cases (14.58 %) had non-keratinizing squamous cell carcinoma (NSCC). 12 patients were diagnosed with stages IV (32.45 %), 16 with stage III (43.24 %), 6 in stage II (16.21 %), and 3 with stage I (8.10 %). As for cancer location, 28 cases (41.79 %) had cancer in post-nasal space, 26 in nasopharyngeal (30.82 %), and 5 in cervical lymph node regions (7.46 %)., as shown in Table 2. A notable fraction of records for age, sample source, cancer stage, and NPC type were categorized as 'Not Available' or 'Not Reported' due to incomplete clinical documentation and constraints in accessing archived tissue information. The exact proportion of missing data is detailed in Table 2.

**Table 2:** General Characteristics of NPC Cases Included in the Study.

Characteristics		Frequency (N)	% (without missing data)
Gender	Female	13/66	19.70 %
	Male	53/66	80.30 %
	NA	4/70	
Age	Mean ± SD	47.24 ± 21.40 years	
	NA	6/70	
Source of sample (malignant tissue)	Postnasal space	28/67	41.79 %
	Nasopharyngeal	26/67	38.82 %
	Cervical lymph node	5/67	7.46 %
	Liver	2/67	2.99 %
	Sinus	1/67	1.49 %
	Tongue or oral cavity	3/67	4.47 %
	Vocal cord polyp	1/67	1.49 %
	Pelvic bone	1/67	1.49 %
	NA	3/70	
Type of NPC	UC	39/48	81.25 %
	NSCC	7/48	14.58 %
	SCC	1/48	2.08 %
	Basaloid squamous cell carcinoma	1/48	2.08 %
	Not reported	22/70	
Cancer stage	Stage I	4/47	8.51 %
	Stage II	7/47	14.89 %
	Stage III	19/47	40.43 %
	Stage IV	17/47	36.17 %
	NA	23/70	
BamHI-W expression by PCR		70	100.00 %

Footnote: NPC, Nasopharyngeal carcinoma; UC, Undifferentiated cell carcinoma; NSCC, Non keratinizing squamous cell carcinoma; SCC, keratinizing squamous cell carcinoma; NA, Not available; N, Number; %, Percentage; SD, Standard deviation.^1^

### Detection of the EBV BamH1-W region in NPC tissue samples

**Figure 1:**
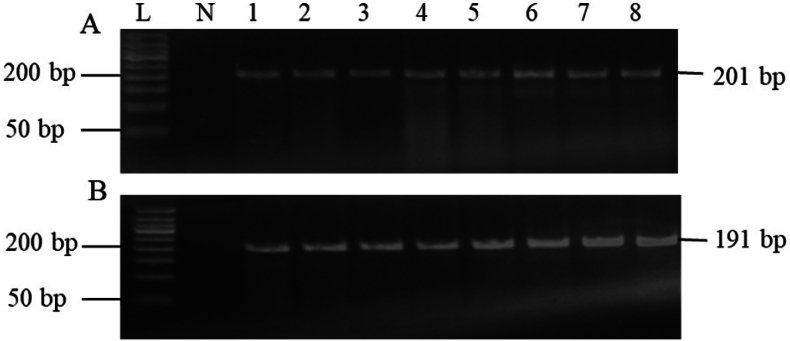
Agarose gel electrophoresis of the PCR product of the beta-actin gene and BamHI-W. (A) beta-actin gene (201 bp) among tissue samples from NPC patients. (B) BamHI-W (191 bp) among tissue samples from NPC patients.L, 50-bp DNA ladder marker; N, negative control (non-template control (NTC)); 1–8 are representative NPC tissue samples.

To evaluate the quality of DNA isolated from FFPE tissue samples, the beta-actin gene was amplified. All 70 samples had the beta-actin band visible (Figure 1A). Additionally, the presence of EBV genomic material in the NPC samples was verified using the BamHI-W gene amplification. BamHI-W was used to detect every sample (Table 2, Figure 1B).

### Detection of the XhoI polymorphism in the LMP1 gene

**Figure 2:**
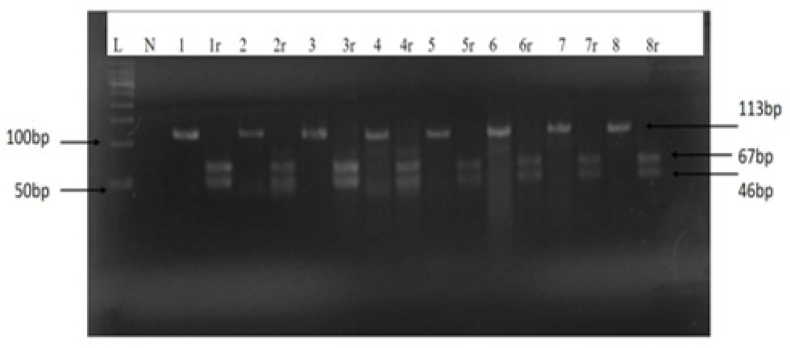
Agarose gel electrophoresis of PCR amplification and XhoI digestion of the EBV LMP1 gene. The 1–8 PCR products (113 bp bands) are representative NPC samples before digestion, and the relevant 1r-8r are representative NPC samples after the XhoI digestion (67 and 46 bp bands). L, 50 bp molecular ladder; N, negative control (non-template control (NTC)).

Using the XhoI restriction enzyme digestion method, 58 out of 70 NPC patient samples (82.86 %) produced two distinct DNA fragments of 67 bp and 46 bp from the 113-bp PCR amplicon, indicating the presence of the wild-type (WT) LMP-1 XhoI variant. Importantly, as shown in Figure 2, none of the samples yielded undigested amplicons corresponding to the mutant-type (MT) variant with restriction site deletion, thus confirming that all examined samples retained an intact XhoI site and no loss-of-site mutations were detected in this cohort."The WT LMP-1 XhoIvariant was found in 58 out of 70 NPC tissue samples and absent in 12 out of 70, was examined in relation to histological type and population characteristics, including NPC type, age, gender, race, and CT scan results indicating disease stage at the last patient follow-up. The findings showed that WT LMP-1 XhoI variant was present in NPC tissue samples from all NPC categories, with non-keratinizing squamous cell carcinoma (NSCC, WHO type II) and undifferentiated carcinoma (UC, WHO type III) having the greatest levels. Nevertheless, there was no statistically significant difference between these two categories (p = 0.669; Table 3). Age and gender did not significantly differ in NPC tissues (p = 0.137 and 0.562, respectively) (Table 3).

**Table 3:** Relationship Between EBV XhoI Detection and the General Characteristics and Histological Features of NPC Patients.

Characteristics		Xhol Frequency N (%)		*p*-value
		Detected n = 58	Not detected n = 12	
Gender	Female	10/13 (76.92) %	3/13 (23.08) %	0.562
	Male	44/53 (83.02) %	9/53 (16.98) %	
	NA	4/70		
Age category	<56 years	28/34 (82.35) %	6/34 (17.65) %	0.137
	≥56 years	24/30 (80.00) %	6/30 (20.00) %	
	NA	6/70		
Source of sample (tissue)	Postnasal space	21/28 (75.00) %	7/28 (25.00) %	0.208
	Nasopharyngeal	24/26 (92.31) %	2/26 (7.69) %	
	Cervical lymph node	4/5 (80.00) %	1/5 (20.00) %	
	Liver	1/2 (50.00) %	1/2 (50.00) %	
	Sinus	1/1 (100.00) %	0/1 (0.00) %	
	Other (Vocal cord polyp/Pelvic bone)	1/2 (50.00) %	1/2 (50.00) %	
	Tongue or oral cavity	3/3 (100.00) %	0/3 (00.00) %	
	NA	3/70		
Type of NPC	Undifferentiated cell carcinoma	32/39 (82.05) %	7/39 (17.95) %	0.669
	Non keratinizing squamous cell carcinoma	7/7 (100.00) %	0/7 (00.00) %	
	Other	2/2 (100.00) %	0/2 (00.00) %	
	Not reported	17	5	
Stages	Stage I	4/4 (100.00)	0/4 (0.00)	0.702
	Stage II	5/7 (71.43)	2/7 (28.57)	
	Stage III	16/19 (84.22)	3/19 (15.78)	
	Stage IV	15/17 (88.24)	2/19 (10.53)	
	NA	23/70		

NA: Not available. N: Number. %: Percentage. NPC: Nasopharyngeal carcinoma. Other2: types of NPC were basaloid squamous cell carcinoma in one case and keratinizing (SCC) type I. ∗Significant *p*-value <0.05 # Fisher exact test. Age was categorized based on median.

### Sequence variation results

**Figure 3:**
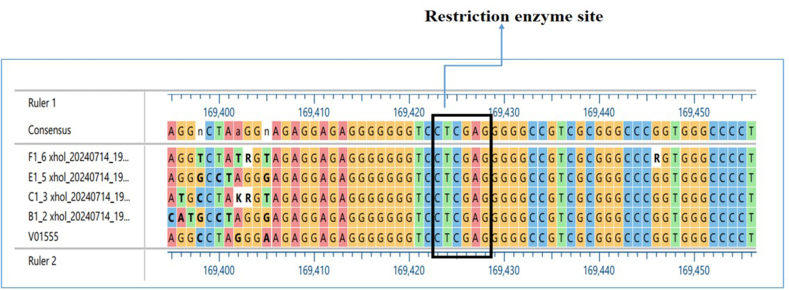
Alignment of B95.8 EBV (V01555) with 4 (F1, E1, C1, B1) Samples from NPC Patients with LMP-1 XhoI Gene.

As illustrated in Figure 3, the XhoI restriction site was identified by aligning the sequencing findings with the B95.8 prototype EBV genome (GenBank accessions no.: V01555.2, from 169,474 bp to 168,163 bp). Figure 3 illustrates the location of the XhoI restriction enzyme site in the DNA sequences, which was found to be between 169,420 and 169,430.

## Discussion

An early diagnosis is essential for NPC patients to have more effective treatment and have a better prognosis.^18^^,^^19^ It is common for patients and physicians to overlook the early symptoms of NPC since they are non-specific and have a limited spectrum.^18^^,^^20^ Despite the advancement of diagnostic tools, including serology, imaging, and fiberoptic nasopharyngoscopy, rarely more than 10 % of newly diagnosed cases of NPC are detected in the early stages.^18^ Biomarkers for early detection, metastasis and recurrence prediction, and therapy monitoring are essential for guiding NPC treatment and improving patient prognosis.^21^ Known as an "all-in-one" oncogene, LMP1 was created throughout the evolution of viruses and serves as a conventional oncogene. It is essential for B cell immortalization and transformation, and it can promote proliferation while inhibiting senescence and apoptosis.^22^, ^23^, ^24^ Previous research has demonstrated a significant association between the expression of LMP1 and the response to treatment, whereby LMP1 promotes metastasis and reduces survival.^25^^,^^26^ Furthermore, LMP1 (+) NPCs was significantly higher survival rates than that of LMP1 (−) NPCs.^25^ The idea that LMP1 acts as an anti-apoptotic factor that influences tumor resistance to anti-tumor drugs is consistent with observations suggesting LMP1 is essential to treatment outcome.^26^, ^27^, ^28^, ^29^ There are many studies that have shown the relationship between EBV and NPC in endemic regions.^8^^,^^17^^,^^30^ According to the WHO report from 2018 in Jordan, the number of individuals with NPC of both sexes was 45 (0.6 % of all types of cancers by primary site).

Additionally, a point mutation in the LMP-1 gene has also been found at nucleotide position G169425T. XhoI, a restriction site in the cytoplasmic N-terminal tail, is deleted as a result of this mutation. Consequently, the XhoI polymorphism is often present in samples from NPC patients but not in samples from healthy individuals.^12^ Using PCR, the current study sought to determine if 70 NPC Jordanian patients had EBV BamHI-W and LMP1 XhoI fragments. The potential utility of these gene biomarkers as a diagnostic biomarker for NPC patients was assessed. Nevertheless, there is currently little data regarding the comparability of different assay types, highlighting the necessity for reliable techniques in the diagnosis of NPC linked to EBV.^31^

Moreover, several earlier studies reported the presence of both LMP-1 and EBNA2 detected in blood, plasma, and EBV-positive cell lines.^21^ The BamHI-W gene is strongly expressed in all forms of EBV latency and functions as a sensitive signal of EBV infection in cells and tissues.^32^ The presence of EBV in the current study was investigated using the BamHI-W gene, which was detected in 100 % of tissue samples from Jordanian NPC patients, indicating that all patients had EBV-DNA. The current finding was consistent with the findings of the previous study, which was conducted by Hadhri-Guiga,^32^ where the repetition of the BamH1-W region was found in postnasal biopsies from 42 NPC patients and 20 controls and demonstrated that 100 % of malignancies were EBV-positive. Despite the variation in studied patients based on gender, age, source of tissue, types of NPC, and stages, all samples were positive for the BamHI-W gene. Therefore, it was concluded that the BamHI-W gene has a high diagnostic value for NPC in Jordanian patients, and it is a distinctive and useful marker in disease diagnosis.

The current study included a higher percentage of men than women, with males accounting for n = 53/66 (80.30 %) and females for n = 13/66 (19.70 %). Comparable to a previous study in Jordan in 2012 (14), which revealed that men were more prevalent than women, with a ratio of 1.45:1. Accordingly, Ayadi (15) indicated that the male-to-female ratio was 2.7:1. A previous study in KSA revealed that NPCs account for 33 % of all diagnosed head and neck cancer, with an age-standardized incidence of 0.08 per 10,000 for females and 0.25 per 10,000 for males.^33^ According to a different study conducted in Malaysia, 124 (78 %) of the 159 NPC patient cases were male, while 35 (22 %) were female. Males are more likely than females to have the malignancy, with a male-to-female ratio of 2.3 to 1 ^34^.

Patients in the current study were mostly between the ages of 50 and 80, with ages ranging from 20 to 80. With a standard deviation of 21.41, the average age of the patients that were enrolled was 47.24 years. Matalka and his colleagues^35^ found that the median age of the patients in a prior study on NPC patients in Jordan was 41 years, with a range of 9–70 years. The age distribution had two peaks: the first one was between 15 and 19 years old, and the second was between 60 and 64 years old. This broad range illustrates how NPC may have an impact on people of many ages. In Tunisian NPC patients, the age range was 11–74 ^8^. While NPC is uncommon in the majority of global populations, it is more common in Southeast Asia and, to a lesser extent, in North Africa and the Arctic.^36^ While there is a little increase in incidence in North Africa between the ages of 10 and 25, there is just one in Southeast Asia at the age of 50 (the majority of cases are between 40 and 60).^36^^,^^37^

The postnasal area is a common source of tissue for NPC, which is the most common cancer to originate in the nasopharynx, most often in the posterior-lateral nasopharynx, though it can also spread to other places. According to Qu et al., metastatic NPCs most frequently occur in the bone, followed by the liver, lung, distant lymph nodes, and brain.^38^ The findings of the current study indicated that the postnasal space is the most common site (n = 28/67, 41.79 %), followed by the nasopharyngeal (n = 26/67, 38.80 %), with other areas of metastasis including the liver, neck, and sinus. Matalka et al.^35^ mentioned that the posterior nasal region was the site of most biopsies, followed by the cervical lymph node, and all cases obtained from the posterior nasal space were positive for NPC. This indicates that the NPC can manifest in various tissues and organs.

As for cancer type, UDC was the most common type of NPC and was observed in 81.25 % (n = 39/48) of the patients in the current study. Non-keratinizing carcinoma (NKCC) was found in 14.58 % (n = 7/48), and other types (basaloid squamous cell carcinoma and KSCC) in 4.17 % (n = 2). UDC is more prevalent, which is consistent with the research currently available on the major histological forms of NPC. Although WHO type I, the KSCC, is primarily prevalent in non-endemic sites, NKC subtypes (WHO types II and III) cause the majority of cases in endemic areas with high prevalence rates of the disease.^39^ Conversely, NKC subtypes have been shown to be associated with EBV infection and are usually positive for EBV.^40^ NPC specimens were classified according to the WHO histological classification^8^: 23 NKC (WHO-2) and 24 UDC (WHO-3). In a study done by Matalka,^35^ the incidence of EBV was investigated only in patients with UDC in the Northern Province of Jordan between 1991 and 2009. Out of 49 cases, 39 specimens were studied, revealing a correlation between clinical variables and EBV results. This indicates that the NKC subtypes (WHO types II and III) are strongly associated with EBV infection, and these results underscore the predominance of UDC in NPC and its significant association with EBV, especially in Jordan, which is considered an endemic region.

Eighty-six percent of the NPC patients in the current study had advanced-stage diagnoses (stages III–IV). This could be explained by the fact that, depending on where they are located and how the nasopharynx is anatomically structured, nasopharyngeal tumors may grow at first without showing any symptoms.^6^ Similarly, in an earlier study, most patients presented with stage III.^41^ This is in agreement with the study done by Ayadi and his colleague, who also reported the highest percentages of EBV appeared in stages III and II.^8^ These results were insignificantly similar to those of other Malaysian studies.^17^ A previous study^42^ reported that stage IV (40.6 %) and stage III (39.1 %) were seen in the majority of NPC patients. However, another study conducted by Pua^43^ showed that, for the first time, EBV DNA levels were considerably greater in NPC patients with stage I illness compared to controls (p < 0.001). Furthermore, stage IV patients with distant metastases had a median level of plasma EBV DNA that was >9 times greater than that of patients without systemic dissemination (p = 0.001), indicating that the detection of plasma EBV DNA may help identify metastatic disease in its advanced stages. El-Sherbieny did a study,^34^ patients with NPC in earlier stages (stages I and II) reported better outcomes than those in later stages (stages III and IV). In predicting the overall survival of the NPC patients, this study showed that the stage at diagnosis was a statistically significant predictive factor. According to this distribution, a sizable fraction of patients received their diagnoses in advanced stages (stages III and IV), which is crucial for treatment planning and prognosis.

As shown in Table 2, there were notable rates of missing data for several key clinicopathological variables, including age, source of sample, cancer stage, and NPC type. This missingness represents an important limitation of the study and may potentially affect the validity of our findings. Such patterns of incomplete data are common in retrospective analyses involving archival hospital tissues. Future studies should aim for standardized and comprehensive data collection practices to enhance analytical robustness and reproducibility.The LMP1 is considered a conventional oncogene, as it is required for B cell immortalization and transformation and can promote proliferation; it is known as an "all-in-one" oncogene that was created during viral evolution.^22^, ^23^, ^24^ In this study, the status of the LMP-1 XhoI mutation was evaluated in nasopharyngeal carcinoma patients. Amplification of the wild-type (WT) LMP-1 XhoI gene was observed in 58 out of 70 NPC tissue samples, while 12 samples did not yield amplification. The occurrence and clinical associations of the WT LMP-1 XhoI variant were analyzed with respect to patient gender, age, tissue origin, NPC histological subtype, and cancer stage; no significant correlations were identified. Furthermore, all analyzed samples lacked the mutant-type (MT) XhoI variant indicative of restriction site loss, paralleling findings previously reported in Tunisian NPC cohorts.^8^ The absence of the XhoI mutation was reported in all Tunisian NPC samples.^8^ On the other hand, as compared to healthy controls, Asian NPC patients had a considerably high XhoI restriction site loss, which has now been identified as a potential tumor biomarker.^44^, ^45^, ^46^ Additionally, the Xhol restriction site was found in all NPC cases from Alaska and some from Caucasian Americans but was absent in NPC cases from Mediterranean Europe and Africa.^47^

In summary, the absence of the LMP-1 XhoI deletion (MT) variant observed in our Jordanian cohort contributes to a growing body of evidence that highlights significant geographic heterogeneity in the molecular genetics of NPC. While the XhoI deletion variant is more prevalent in East and Southeast Asian populations,^48^, ^49^, ^50^, ^51^ our findings support reports of its rarity in the Middle East. This emphasizes that molecular biomarker patterns in NPC are highly region-dependent and should be interpreted within their specific geographical context. These region-specific genetic differences are increasingly recognized as critical factors influencing both the pathogenesis and clinical management of NPC, as highlighted by large-scale sequencing and case–control studies.^48^, ^49^, ^50^, ^51^, ^52^, ^53^ The present findings build upon earlier work in North Africa, all of which underscore the absence or rarity of LMP-1 XhoI deletion in non-Asian contexts.^48^, ^49^, ^50^, ^51^ Therefore, our results not only reinforce the importance of geographic qualifiers in the reporting of molecular NPC data but also support the paradigm that biomarker utility and underlying viral genetic diversity in NPC require local population-based assessment for effective clinical application.

The findings confirm 100 % positivity for EBV BamHI-W DNA in tumor tissue, supporting the virus's oncogenic role in NPC within the study population. However, consistent with current literature and reviewer suggestions, tissue-based EBV detection is not recommended for early clinical diagnosis. Instead, circulating plasma EBV DNA is widely recognized for its diagnostic and prognostic advantages, permitting detection of NPC at much earlier stages and allowing ongoing monitoring of disease progression and treatment response.^54^^,^^55^ In addition, the absence of LMP-1 XhoI polymorphism (MT variant) and its lack of association with demographic or clinical characteristics in this cohort further affirm its limited diagnostic and prognostic value in NPC in Jordan and neighboring regions.

## Conclusions

This study demonstrates that EBV is present in all NPC cases, as evidenced by universal detection of the BamHI-W gene. The WT LMP-1 XhoI variant was detected in 82.86 % of samples, while the MT variant was absent in all cases, a result validated by Sanger sequencing. These findings suggest that, despite EBV's central pathogenic role, LMP-1 XhoI mutation does not serve as a reliable diagnostic and prognostic marker for NPC in this cohort. BamHI-W gene detection proves to be a more consistent indicator of EBV presence within tumor tissues; however, its diagnostic value for early clinical detection is limited. Therefore, the study reinforces the principal importance of plasma EBV DNA quantification as a superior diagnostic and prognostic modality in NPC. The absence of MT LMP-1 XhoI in Jordanian samples affirms the region-specific landscape of NPC-associated viral genetic variants, underscoring the need for localized molecular epidemiological research. Collectively, these outcomes indicate that LMP-1 XhoI polymorphism has limited clinical applicability within this regional context. Future research should prioritize blood-based EBV markers and explore additional viral and host genetic determinants to advance NPC diagnosis and prognosis.

## Data availability statement

All of the data related to the current study has been reported in the manuscript. There is no data has been deposited in a public repository or any other database.

## Ethical approval

The ethical approvals were obtained from Institutional Board Committees (IRB) (IRB 33/166/2024) from Zarqa University and from the KAUH-IRB committee (13/2/157).

## Consent

All patients provided written informed consent to participate in the study. The study was performed in accordance with the relevant institutional and national guidelines and regulations.

## Authors contributions

Conceptualization, Mai Abusalah and KA-Q; software, Manal Abusalah and MAR; validation, NA and KA-Q; formal analysis, YR, AR and AA-H; investigation, YR and AA-H; resources NA and Mai Abusalah; data curation, NA and Mai Abusalah; writing—original draft preparation, YR, AA-H, YAS, M. D, MAR, Mai Abusalah and KA-Q; writing—review and editing, NA and Mai Abusalah; visualization, NA; supervision, Mai Abusalah and KA-Q. All authors have read and agreed to the published version of the manuscript. All authors have critically reviewed and approved the final draft and are responsible for the content and similarity index of the manuscript. All authors have critically reviewed and approved the final draft and are responsible for the content and similarity index of the manuscript.

## Source of funding

This research did not receive any specific grant from funding agencies in the public, commercial, or not-for-profit sectors.

## Conflict of interest

The authors have no conflict of interest to declare.
